# Matrixlysis, an improved sample preparation method for recovery of Mycobacteria from animal tissue material

**DOI:** 10.1371/journal.pone.0181157

**Published:** 2017-07-19

**Authors:** Christoph Leth, Ashok Varadharajan, Patrick Mester, Marlis Fischaleck, Peter Rossmanith, Friedrich Schmoll, Maria Fink

**Affiliations:** 1 Austrian Agency for Health and Food Safety, Institute for Veterinary Disease Control, Moedling, Lower Austria, Austria; 2 University of Applied Sciences FH Campus Wien, Vienna, Austria; 3 Laboratory for Functional Genome Analysis (LAFUGA), Gene Centre, LMU Munich, Munich, Germany; 4 Christian Doppler Laboratory for Monitoring of Microbial Contaminants, University of Veterinary Medicine, Vienna, Austria; Indian Institute of Technology Delhi, INDIA

## Abstract

*Mycobacterium caprae*, a member of the *Mycobacterium tuberculosis complex*, is the main causative agent of bovine tuberculosis in alpine regions. Bacterial culture is the gold standard in bovine tuberculosis diagnostic but takes up to twelve weeks. This increases the time and costs for stocks affected with bovine tuberculosis. Hence this study focused on the implementation of a fast and precise mycobacterial detection method and compared it with currently used methods. Matrix lysis is a chemical lysis using high concentrations of urea to solubilize bovine and red deer tissue and was used to detect even smallest amounts or non-visible lesions of mycobacteria. A total of 64 samples collected from 44 animals (37 red deer and 7 cattle) were tested by Matrix lysis. Forty-three of these samples were used for *Mycobacterium tuberculosis complex* detection by quantitative PCR and other 21 for subtyping the genetically different variants of *M*. *caprae*. Furthermore, three Matrix lysis samples were used for Next Generation Sequencing. Our results confirm that Matrix lysis is a fast and precise method for detecting *Mycobacterium tuberculosis complex* in native tissue samples. However, at the moment it reaches its limits when the samples were analyzed by Next Generation Sequencing and RD4 subtyping.

## Introduction

*Mycobacterium caprae* and *M*. *bovis*, both member of the Mycobacterium Tuberculosis Complex (MTC), are the causative agents for bovine tuberculosis (bTB)[1]. While *M*. *bovis* is a long known member of the MTC, *M*. *caprae* was primarily described by Aranaz *et al*. in 1999 in goat samples in Spain [2]. Although Austria is listed as bovine tuberculosis free, infections of cattle and red deer with *M*. *caprae* are still a serious problem in the western parts of Austria. Currently bacterial culture is the "gold standard" in detecting MTC and investigation of up to twelve weeks are necessary to obtain an unambiguous result. An alternative provides PCR, but due to its relatively high possibility of false negative results, caused by the uneven distribution of mycobacteria in tissue and small amounts which can be used for PCR, molecular diagnostic methods still is an ineligible method to use as final result. During this study we evaluated a preparation method (Matrixlysis) to eliminate both of the above mentioned methods’ disadvantages and create a fast and precise sample preparation and detection method. Previously, Mann *et al*. developed a model system in which different pig tissues were spiked with *Listeria monocytogenes* and the so called method ML was tested with different Lysis buffers according to the tissue solubilisation characteristics and the intended detection method [3,4]. Based on this model system we adapted ML for quantifying gram positive mycobacteria in naturally infected tissue material. Diagnostic difficulties of TB presenting non visible lesions (NVL) were described by Gabier-Widén *et al*. and de Lisle *et al*. in 2009 [5,6]. They focused on *M*. *bovis* infections causing no macroscopically visible lesions and the resulting problems of inaccurate TB diagnosis. Because ML is not limited to the quantity of tissue, large amounts of tissue can be used to avoid false negative results in case of very small or non visible lesions. An alternative to ML could be the previously developed technique using magnetic beads described by Fell *et al*. [7]. This technique relies on mechanical tissue disruption followed by target specific DNA capture, using biotinylated oligonucleotides and magnetic beads. Another magnetic beads approach was described by Stewart *et al*. [8] focusing on Immunomagnetic separation of the whole bacterium by using antibody coated beads. In this case, mycobacteria were still viable after immunomagnetic separation and therefore could be used for bacterial cultivation. To differentiate between the known MTC species the GenoType MTBC line probe assays (Hain Lifesciences GmbH, Nehren, Germany) or qPCR in accordance to Reddington *et al*. [9] can be used. Because both methods rely on a single SNP, GenoType MTBC on a T to G substitution on position 1311 in the *gyrB* sequence [10–12] and Reddington *et al*. [9] on a C to T substitution at position 690 in the H37 Rvl*epA* sequence, they need purified *M*. *caprae* DNA which can only be provided by cultivation of the bacteria. To differentiate between the three alpine *M*. *caprae* subtypes mycobacterial interspersed repetitive-unit-variable-number tandem-repeat (MIRU- VNTR) typing is used [13,14]. Spoligotyping cannot be recommended for the subtyping of the three alpine *M*. *caprae* subtypes due to its limited discrimination [13]. A new method, previously described by Domogalla *et al*. and adapted by Rettinger *et al*. used the Region of difference four (RD4) to differentiate between three *M*. *caprae* subtypes ‘Allgäu’, ‘Karwendel’ and ‘Lechtal’ which were named according to their geographical appearance [15,16]. While they showed equal sequences in the remaining RDs by whole genome sequencing different RD4 deletions can be used to identify *M*. *caprae* and its three alpine subtypes by PCR. Specific primers use the different deletion patterns of the three subtypes to differentiate between them. *M*. *caprae* of the Allgäu subtype contains the whole RD4, the Karwendel subtype a 5kb deletion and the Lechtal subtype a 38kb deletion [15]. This is in contradiction to former differentiation models using the complete absence or presence of the RD4 as distinguishing criterion for species identification of *M*. *bovis* and *M*. *caprae*. This hypothesis was disproved by Domogalla *et al*. and Rodrigues-Campus *et al*. [15,17].The aim of this study was to adapt the suitability of ML for detection of MTC from bovine and red deer lymph nodes of naturally infected lymph nodes. The method ML was compared to the direct-PCR approach, bacterial cultivation and homogenate PCR approach currently used in routine diagnostics of many diagnostic laboratories. Additionally, ML samples were subtyped by the RD4 method and several ML samples sequenced from methods by whole-genome sequencing without bacterial cultivation.

## Material and methods

### Ethics statement

Based on Austrian federal regulations bTB in cattle and red deer is an eradicable disease under mandatory surveillance and monitoring. Suspicious samples are submitted at the National Reference Laboratory for confirmation of bTB diagnosis by bacteriological culture and PCR. Analysis of animal specimens was therefore carried out within an official context, meaning that no animals were killed for the purposes of this research project and ethical approval was not necessary.

### Sample material

Twenty five red deer animals delivering 43 samples from different tissue, which were identified as *M*. *caprae* within the ERA-Net EMIDA project "Tb in alpine wildlife" or by different Austrian red deer surveillance programmes were used in this study (Table 1) [18]. All 25 red deer animals and one cattle were used for quantification of mycobacteria in native sample material using different methods and compared by results from bacteriology. Eleven out of these 25 red deer animals (red deer animal 16 to 26) composed a complete sample set of both medial retropharyngeal lymph nodes (pool A), tracheobronchial- mediastinal- and mesenteric lymph nodes (pool B) as also any other suspicious tissue or lymph nodes material with macroscopically visible lesions (pool C). The sample sets of the other 14 red deer animals (red deer animals 1 to 14) which were hunted for Austrian red deer surveillance programmes and the cattle (cattle 15) which was slaughtered in accordance to the Austrian RinderTbV composed different sample materials with macroscopically visible lesions only (Table 1).

**Table 1:** Sample material for quantitative analysis.

Sample No.	Host species	Sex and Age	Sample material	Culture result
1	Red deer	stag II	2 Re	+
2	Red deer	n.d.	Re	+
3	Red deer	n.d.	Mes,Tra,Lu	+
4	Red deer	n.d.	Re	+
5	Red deer	n.d.	Re	+
6	Red deer	n.d.	Re	+
7	Red deer	n.d.	2 Re	+
8	Red deer	n.d.	2 Re	+
9	Red deer	n.d.	Mes	+
10	Red deer	n.d.	Tra, Med	+
11	Red deer	n.d.	Re	+
12	Red deer	n.d.	Re	+
13	Red deer	hind	Par, Li, Man, Re, Tra, Med,	+
14	Red deer	hind	Re	+
15	Cattle	female,13years	Med, Lu	+
16	Red deer*	stag II	A and B	unknown
17	Red deer*	stag II	A, B, C(Re)	unknown
18	Red deer*	hind > 2y	A, B, C(Re)	unknown
19	Red deer*	stag II	A, B, C(Lu, Tra)	unknown
20	Red deer*	stag II	A, B, C(Par)	unknown
21	Red deer*	stag II	A, B	unknown
22	Red deer*	stag III	A, B	unknown
23	Red deer*	hind > 2y	A, B, C(Med, Tra)	unknown
24	Red deer*	stag II	A, B	unknown
25	Red deer*	stag I	A, B, C(Re)	unknown
26	Red deer*	stag III	A, B	unknown

Re, one medial retropharyngeal lymph node; 2Re, both medial retropharyngeal lymph nodes; Mes, mesenteric lymph node; Med, mediastinal lymph node; Tra, tracheobronchial lymph node; Lu, lung tissue; Li, liver lymph node; Man, mandibularis lymph node; Par, parotid lymph node

*, free ranging red deer animals hunted within the EMIDA ERA-Net project; A, pool A containing both medial retropharyngeal lymph nodes; B, pool B containing TraMedMes;C, pool C is determined; stag III, male between 1 and 4 years; stag II, male between 5 and 9 years; stag I, male older than 9 years; hind > 2y, female older than 2 years; hind < 2y, female younger than 2 years; n.d., not defined

Sample material containing macroscopically visible lesions from additional 12 *M*. *caprae* confirmed red deer animals hunted for Austrian red deer surveillance programmes (red deer animal 27 to 38) and five *M*. *caprae* confirmed cattle slaughtered in accordance to the national legislation RinderTbV 2008;current status (cattle 39 to 44) were used for ML RD4 subtyping and three for whole genome sequencing by the Illumina sequencing technique.

#### Sample preparation and quantitative PCR (Fig 1)

Enrichment of bacteria was performed by ML which proceeds in three steps: 1. the homogenization of the tissue material; 2. the chemical lysis of the matrix (tissue) and; a washing step (3.) of the pelleted bacteria was necessary to remove any left lysis buffer. The resulting pellet was dissolved in ATL buffer of the DNA extraction kit. The homogenate qPCR method used the same starting material. Therefore an aliquot of step 1 of ML was taken away after homogenization. Precipitation of solid material was performed by centrifugation and the resulting pellet is dissolved in ATL buffer of the same DNA extraction kit. Direct qPCR is the only method which directly used the tissue material. Therefore a very small piece of tissue was taken up in Lysis buffer of another DNA extraction kit. Due to the robust and complex structure of the mycobacteria wall, a mechanical disruption, called bead beating, was performed for all three methods in order to release any nucleic acid from the mycobacteria. QPCR of all samples was performed by the same qPCR kit.

**Fig 1:**
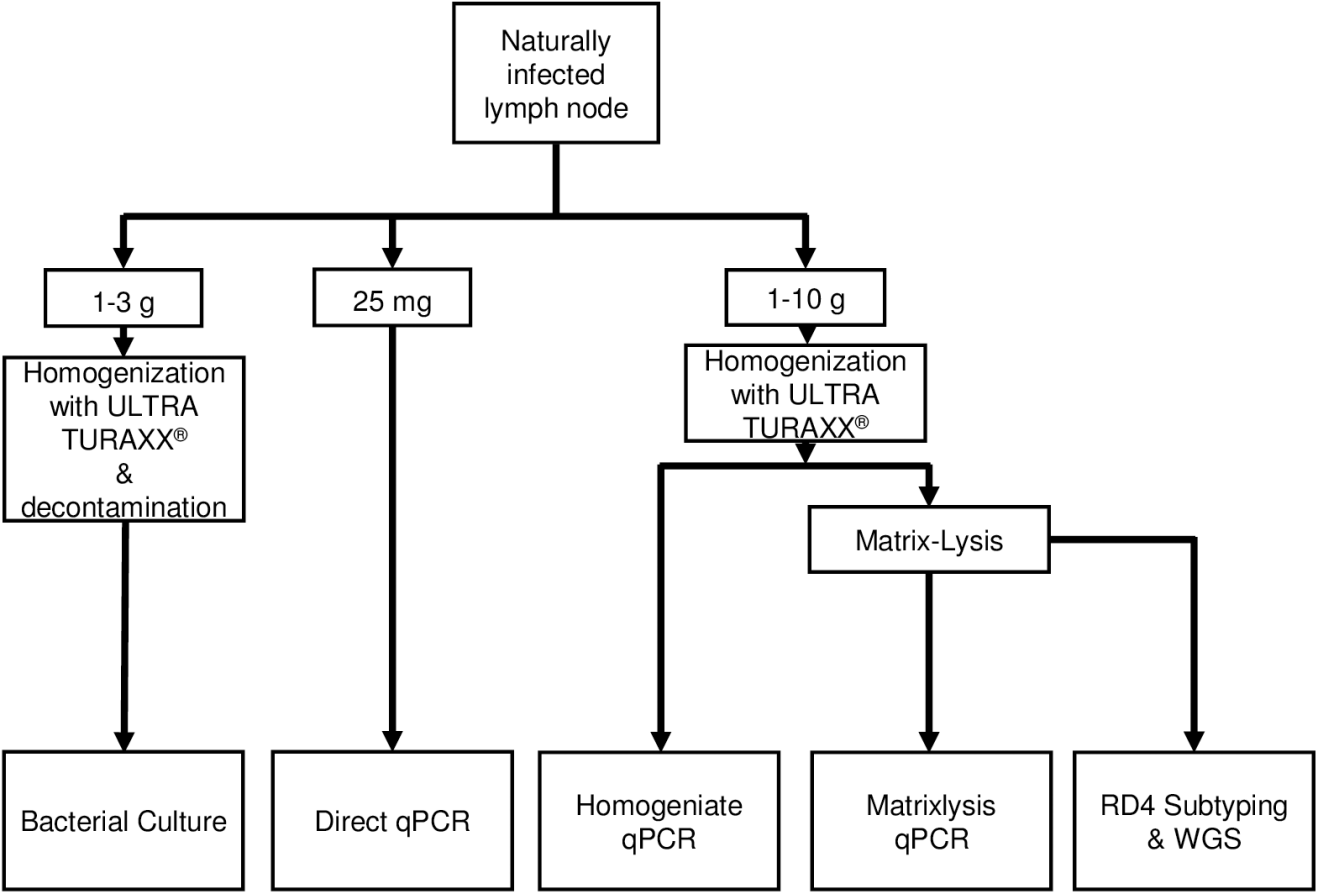
Workflow of samples.

### DNA extraction for direct qPCR

25 milligrams (mg) of suspicious lesions or visible lesion containing sample material was cut out with a scalpel from the tissue or lymph nodes and suspended in 200 μl Tissue Lysis Buffer of the High Pure PCR Template Preparation kit from Roche, Rotkreutz, Switzerland. Bead beating was performed by transferring the lysis material into a 2 ml safe lock centrifugation tube (Eppendorf, Hamburg, Germany) containing a 5 mm stainless steel bead (Qiagen, Hilden, Germany). For mechanical burst of the mycobacterial cell wall, the tube was shaken 2 times for 3 min at 30 Hertz (Hz) using the Mixer Mill 301 (Retsch, Haan, Germany). The obtained suspension was used for proper DNA extraction as recommended by the manufacturer of the High Pure DNA Template Preparation kit.

### Matrixlysis (ML)

For bacterial recovery up to ten grams (g) of frozen tissue material was homogenized in five millilitre (ml) Solution A (0.25 M Sucrose, 1 mM EDTA, 0.05 M Tris pH 7.6; 0.1% (w/v) Savinase GTT24) using the ULTRA-TURRAX® Tube Drive Workstation (IKA^®^, Staufen, Germany). Tissue material which was too large to fit into the DT-20 Tube (IKA^®^, Staufen, Germany) was cut with a scalpel and treated equally. If necessary, 5 ml aliquots were pooled in a 50 ml screw cap tube (50ml SuperClear^TM^ Centrifuge Tube with plug style cap, VWR, Radnor, USA). The homogenized material containing 5, 10 or 15 ml was filled up to 50 ml with phosphate buffered saline (PBS), incubated for 30 min at 37°C in a shaking water bath (1003, GFL, Burgwedel, Germany) and centrifuged for 30 min at 3220 x g (Rotina 420, Hettich, Tuttlingen, Germany). The supernatant was discarded and the pellet was resuspended in 45 ml Lysis Buffer (8 M Urea, 1% (v/v) SDS, 1 x PBS). After incubating for 30 min at 37°C in a shaking water bath and centrifugation for 30 min at 3220 x g, the supernatant was discarded and the pellet was resuspended in 40 ml Washing Buffer (1 x PBS, 0.35% (v/v) Lutensol AO-7). Again, the samples were incubated for 30 min at 37°C in a shaking water bath, centrifuged for 30 min at 3220 x g and the supernatant was discarded. The pellet was resuspended in 1 ml PBS, transferred into a 2 ml reaction tube (Eppendorf, Hamburg, Germany) and centrifuged for 5 min at 8000 x g (5415R, Eppendorf, Hamburg, Germany). This final washing step was repeated once.

### DNA extraction for ML qPCR and homogenate qPCR

For ML qPCR the bacteria pellet resulting from ML was resuspended in 180 μl Lysis Buffer ATL of the kit DNeasy^®^Blood&Tissue Kit from Qiagen, Hilden, Germany. For the homogenate qPCR, 1 ml of homogenized sample material (Fig 1) was centrifuged for 25 min at 16000 x g; the supernatant discarded and the pellet resuspended in 180 μl Lysis Buffer ATL of the DNeasy^®^Blood&Tissue Kit. To perform bead beating, the lysed sample material was transferred into a 2 ml safe lock centrifugation tube (Eppendorf) containing a 5 mm stainless steel bead (Qiagen). For mechanical burst of the mycobacterial cell wall the lysis material was treated as described by direct qPCR using the Mixer Mill 301. The obtained suspension was used for proper DNA extraction as recommended by the manufacturer of the DNeasy^®^Blood&Tissue kit.

### DNA extraction for RD4 subtyping and whole genome sequencing

For RD4 subtyping and whole genome sequencing the matrixlysis pellet was suspended in corresponding cell suspension solution of the Gentra Puregene Yeast/Bact. Kit (Qiagen, Hilden, Germany) and DNA extraction performed as described.

### QPCR (quantitative PCR) or real-time PCR

QPCRs were performed with the artus^®^ M. tuberculosis TM qPCR Kit (Qiagen/Deutschland), according to the manufacturers protocol. Detection was performed on the 7500 Fast Real-Time PCR System (Applied Biosystems, Waltham, USA).

### Artificial inoculation of bovine lymph nodes

In order to evaluate the applicability of ML for MTC detection, artificial contamination experiments with bovine lymph nodes were performed. For these experiments, five PCR negatively-tested bovine lymph nodes (5g each) were spiked with 100μl of a *Mycobacterium bovis Bacillus Calmette-Guérin* (BCG). Therefore, the *M*. *bovis BCG* was grown in Middlebrook 7H9 liquid media containing Tween 80 (0.05%) for three to four weeks. The spiked lymph nodes were processed with ML and subsequently subjected to DNA-extraction and qPCR, while the original five *M*. *bovis BCG* inocula aliquots were directly subjected to DNA-extraction and qPCR without extra processing.

### Pathoscoring of naturally infected samples

A bTB lesion score (pathoscore) was defined for lymph nodes and lung tissue in accordance to Ballesteros et *al*. [19]. It considers the distribution and the intensity of the lesions. Lesions of a size less than 1 cm are classified as "A" lesions and lesions exceeding 1 cm as "B" lesions. Summarizing, pathoscore 0 always determines a NVL sample; samples of pathoscore 1 and 2 only contain type "A" lesions and samples of pathoscore 3 or higher contain lesions of type “B”.

### Bacteriology (Fig 1)

For bacteriological cultivation, two to three grams of pathologically suspicious tissue material was homogenized in five ml NaCl solution (0.9%) using the IKA Ultra-Turaxx^®^ Tube Drive Workstation (IKA^®^, Staufen, Germany). The suspension was decontaminated with 1% N-Acetyl-L-Cystein-NaOH solution and neutralized with 20 ml phosphate buffer (pH 6.8) as recommended by the World Organization for Animal Health (OIE) (2012) Manual [20]. The solution was centrifuged for 20 min at 3300 x g and the suspension discarded. The suspension pellet was plated on two growth media—Lowenstein-Jensen with Glycerin and PACT (polymyxin B, amphotericin B, carbenicillin and trimethoprim) and Stonebrink with PACT (both purchased from Heipha) using swabs. Incubation was performed at 37°C for 12 weeks and bacterial growth was frequently screened. *M*. *caprae* species identification was performed by reversed line blotting (Geno Type® MTBC, HAIN Life Science, Germany).

#### Sample preparation RD4 subtyping and next generation sequencing (Fig 1)

For RD4 subtyping and whole genome sequencing DNA extraction was performed using the GentraPuregene Yeast/Bact. Kit as described by Broeckl *et al*. [21]. Bacterial colonies were scraped off the solid Loewenstein-Jensen or Stonebrink solid growth media and suspended and washed in 1 ml PBS by vortexing. After centrifugation for 6 min at 15000 x g (5415R, Eppendorf, Hamburg, Germany) the samples were resuspended in 600 μl Cell Suspension Solution. Cell pellets resulting from ML were directly suspended in 600 μl Cell Suspension Solution. Three μl Lytic Enzyme were added and the samples were inverted for 25 times in a 1.5 ml centrifugation tube (Eppendorf, Hamburg, Germany). After incubation for 30 min at 37°C and centrifugation for 6 min at 15000 x g the supernatant was discarded and the pellet was resuspended in 450 μl Cell Lysis Solution and 15 μl Proteinase K. Lysis was performed at 57°C and 600 rpm on a shaking Thermocycler (Thermomixer Comfort, Eppendorf, Hamburg, Germany) overnight. To stop proteolytic proteinase K activity, the samples were incubated on ice for 1 min, 160 μl Protein Precipitation Solution were added and the samples were mixed for 20 seconds using a vortex. After centrifugation for 6 min at 15000 x g, the supernatant was transferred into a new 1.5 ml reaction tube (Eppendorf, Hamburg, Germany) containing 600 μl Isopropyl alcohol (100%) and 1.2 μl Glycogen. The samples were mixed gently by inverting 50 times and centrifuged for 15 min at 15000 x g. The supernatant was discarded and the pellet was resuspended and washed in 700 μl Ethanol (70%(v/v)). The samples were centrifuged for 10 min at 14000 x g and air dried for up to 10 min. The dry pellet was resuspended in 100 μl DNA Hydration Solution and incubated at 65°C for one hour.

### RD4 subtyping

RD4 Subtyping was performed as developed and described by Domogalla *et al*. and Rettinger *et al*. with adaptions as following: All reactions were carried out as simplex in a total volume of 25 μl, each reaction contained 0.625 U of HotStarTaq® DNA Polymerase (Qiagen, Hilden, Germany), 0.5 μM Primer (for detailed Primer sequences see Domogalla *et al*.), primers flanking the 38kb deletion annealed at 60°C. After PCR amplification, the PCR products were separated by gel electrophoresis in 1% agarose gels in Tris-borate EDTA (1 x TBE) buffer at 120V for 40 min and visualized using PeqGreen from Peqlab (5μl/100ml agarose gel).

### Next generation sequencing

Sequencing libraries were constructed from the DNA of three samples that were subjected to the Matrix Lysis. Each sample with 325 ng of DNA was fragmented by sonication with a M220 Ultrasonicator (Covaris, MA, USA) to a medium length of 250 bp. Fragmented DNA were then used to construct sequencing libraries with the single-stranded Next generation sequencing (NGS) Library Preparation Kit (Swift Bioscience, Ann Arbor, USA) according to the manufacturer`s instructions and Dual Indexing was performed with the length of 8 basepairs barcodes each. Finally, the Next Generation Sequencing was done on an Illumina HiSeq 1500 (Illumina, San Diego, USA) sequencer in a paired-end mode with a read length of 100 bp in each direction. Raw sequencing data from the sequencer was obtained as FASTQ format. In order to reassign the reads to their sample origin, raw data were de-multiplexed based on their respective dual barcodes with the Je's Illumina-illu [22] tool. Only those reads that fulfil strict quality criteria for both index reads are selected for further analyses. These criteria are a complete index sequence without mismatch and a miminum quality of Q30. The resultant reads were mapped against the assembly of both *M*. *caprae* [21] and *Bos taurus (UMD3*.*1*.*1)* using the BWA mem [23]. Percentage of duplicated reads and summary of the mapping statistics was then estimated using samtool's rmdup [24] and flagstat [24] module respectively.

## Results

### Artificial inoculation of bovine lymph nodes

The results of this evaluation of ML applicability showed that *M*. *bovis Bacillus Calmette-Guérin* was reproducibly recovered from 5g lymph node matrix with an efficiency of 24%. The average ct-value was 25.7 for the spiked lymph nodes and 23.4 for the *M*. *bovis Bacillus Calmette-Guérin* culture. These results are in good agreement to the efficiencies obtained by Mayrl *et al*. [4] and demonstrate the applicability of the ML for lymph node tissue. Table 2 shows the results of 5 x 5g bovine lymph node tissue, spiked with Mycobacterium bovis BCG and ML processed. Ct-values were compared with the untreated BCG inoculum.

**Table 2:** qPCR results of artificially spiked lymph nodes.

Replicate Nr.	Control (Ct)	Ct after Matrixlysis
I	23.8	24.9
II	24.1	26.9
III	23.3	25.9
IV	21.9	24.7
V	23.5	25.9
Avg	23.4	25.7
RSD*	0.86	0.90
Recovery		24%

* Relative standard deviation (RSD)

### ML qPCR in comparison to bacterial culture, direct qPCR and homogenate qPCR

**Fig 2:**
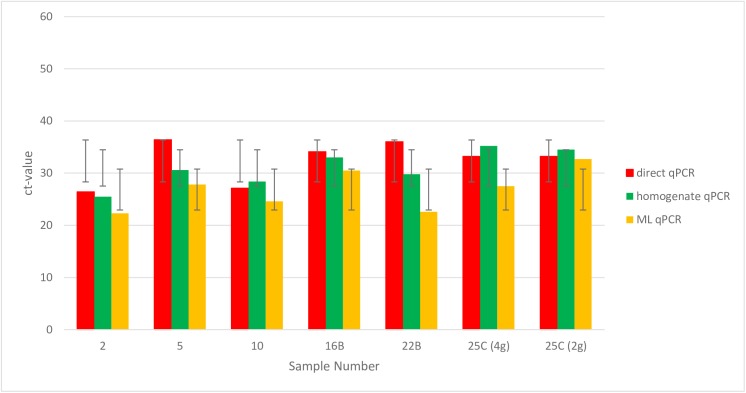
Statistical analysis showing ct-values and standard deviations of sample sets 2, 5, 10, 16B, 22B and 25C. ML of sample 25C was performed twice using 2 gram and 4 gram tissue material, respectively.

As primary step in quantifying naturally infected tissue material, bacterial culture positive samples of animals 1 to 15 (Table 1) were used to test if ML is capable for subsequent DNA extraction and qPCR and to determine the sensitivity of ML qPCR in comparison to bacteriology, the "gold standard" in Tb diagnostic. Therefore, the samples were analysed by direct qPCR, homogenate qPCR, and ML qPCR and compared to the positive results from bacteriology. For ML qPCR, a ct-value was obtained for all 15 samples and therefore the sensitivity was determined as least as high as for bacteriology. Both methods, direct qPCR and the homogenate qPCR, detected MTC in 13 of 15 positive animals and were therefore less sensitive than ML and the bacterial culture (Table 3, Fig 2). When comparing the ct-values of the different methods, ML qPCR showed in 12 cases the lowest ct-value and therefore the highest sensitivity of all quantitative methods (Table 3). Only red deer sample 1 and 9 showed a lower ct-value by direct qPCR indicating that in these two cases the 25 mg contained the optimal "lesion-containing" material (Table 3). Regardless of these two cases, the differences in the ct-values between ML qPCR and direct qPCR varied from 0.5 to 8.6 ct-values. The average delta ct-value was 3.13 lower than for direct qPCR (not indicated). The differences in the ct-values between ML qPCR and the homogenate qPCR varied also from -1.0 to 6.4 which was unexpected as the starting material for both methods was the same and one would expect a constant relation between the ct-values of both methods. One explanation might be that mycobacteria are not evenly diluted in the homogenized tissue solutions because they form lumps. This might also explain why the ct-value of the homogenate qPCR of red deer animal 7 was one ct-value lower than for the ML qPCR. In this case the 1 ml aliquot of the homogenized sample material used for the homogenate qPCR method "accidentally" contained a lump of mycobacteria counting twice of the amount than used for ML (delta ct 1).

**Table 3:** ML qPCR was compared to direct qPCR, homogenate qPCR and bacteriology.

Samp. No.	Host species	Lymph node/ tissue		Culture	qPCR results (Ct)			weight of lymph node for ML [g]	Pathoscore
					Direct	Homogenate	ML		
1	Red deer	2 Re		+	38,5	41,5	40,2	4,2	3
2	Red deer	Re		+	26,4	25,5	22,3	2,4	3
3	Red deer	Mes,Tra,Lu		+	no ct	33,9	31,2	2,7	3
4	Red deer	Re		+	no ct	no ct	36,0	1,3	1
5	Red deer	Re		+	36,4	30,6	27,8	2,7	3
6	Red deer	Re		+	32,2	32,5	29,1	2,4	3
7	Red deer	2 Re		+	36,4	35,4	36,4	2,3	2
8	Red deer	2 Re		+	32,7	32,2	29,4	2,2	3
9	Red deer	Mes		+	28,4	37,0	33,1	1,0	3
10	Red deer	Tra, Med		+	27,1	28,4	24,6	2,3	3
11	Red deer	Re		+	40,0	no ct	34,1	2,5	2
12	Red deer	Re		+	37,0	41,9	35,6	2,3	2 or 3*
13	Red deer	Par, Li, Man, Re, Tra, Med,		+	35,8	36,3	34,5	5,0	3
14	Red deer	Re		+	34,0	37,4	33,5	2,1	1
15	Cattle	Med, Lu		+	32,3	33,9	28,6	4,1	2
No. of positive animals				15 / 15	13 / 15	13 / 15	15 / 15		

Re, one medial retropharyngeal lymph node; Mes, mesenteric lymph node; Med, mediastinal lymph node; Tra, tracheobronchial lymph node; Lu, lung tissue; Li, liver lymph node; Man, mandibularis lymph node; Par, parotid lymph node; 2Re, both medial retropharyngeal lymph nodes; ML, Matrixlysis

* a more precise pathoscore determination not possible

After this pre-test with only culture-positive sample material, 28 samples of 11 red deer animals (red deer animal 16 to 26, Table 1) from the ERA-Net Project were examined (Table 4, Fig 2). These red deer animals comprised a whole sample set, which was already predefined: pool A contained both retropharyngeal lymph nodes, pool B the tracheobronchial-, mediastinal- and mesenterial lymph nodes and in cases of red deer animals 17, 18, 19, 20, 23 and 25 any further visible lesion containing tissue material as indicated in sample pool C (Table 4). Not all red deer animals were confirmed positive by bacteriology.

**Table 4:** ML qPCR of 28 sample materials from 11 red deer animals compared to direct qPCR, homogenate qPCR and bacteriology.

Samp. No.	Host species	Lymph node/tissue		Culture	qPCR results (Ct)			weight of lymph node for ML [g]	Pathoscore
					Direct	Homogenate	ML		
16	Red deer	- A		+	33,8	38,0	37,6	2,3	4
		- B		+	34,1	33,0	30,5	2,2	4
17	Red deer	- A		-	no ct	no ct	no ct	5,0	0
		- B		-	no ct	no ct	no ct	3,1	0
		- C(Re)		-	no ct	39,1	35,1	2,0	1
18	Red deer	- A		-	no ct	no ct	no ct	4,9	0
		- B		-	no ct	no ct	no ct	2,9	0
		- C(Re)		+	no ct	33,2	28,9	5,7	2
19	Red deer	- A		-	no ct	no ct	no ct	6,7	0
		- B		-	no ct	no ct	no ct	2,0	0
		- C(Lu &Tra)		+	32,2	35,3	29,4	5,5	3,4
20	Red deer	- A		-	no ct	no ct	29,5/33,8	2,6/7,2	0
		- B		-	no ct	no ct	36,1	3,4	0
		- C(Par)		+	34,2	38,4/36,5	38,2/34,9	2,2/6,3	3
21	Red deer	- A		+	no ct	36,3	37,6	2,8	1
		- B		+	no ct	no ct	no ct	4,4	0
22	Red deer	- A		+	25,4	36,7	30,1	2,2	3
		- B		-	36,0	29,8	22,6	8,5	0
23	Red deer	- A		-	no ct	no ct	37,0	2,9	0
		- B		-	no ct	no ct	no ct	2,1	0
		- C(Med,Tra)		+	36,4	no ct	32,0	2,4	3,1
24	Red deer	- A		+	40,0	no ct	35,2	10,0	3
		- B		-	no ct	no ct	39,2	1,5	0
25	Red deer	- A		+	no ct	no ct	44,4	2,3	1
		- B		-	no ct	39,6	35,1	4,9	0
		- C(Re)		+	33,2	34,5/35,2	32,7/27,5	2,0/4,0	3
26	Red deer	- A		+	36,0	36,4	31,5	3,6	3
		- B		-	no ct	no ct	32,8	3,2	0
No. of positive lymph nodes				13 / 28	10 / 28	12 / 28	20 / 28		
No. of positive animals				10 / 11	8 / 11	9 / 11	11 / 11		

Re, one medial retropharyngeal lymph node; Mes, mesenteric lymph node; Med, mediastinal lymph node; Tra, tracheobronchial lymph node; Lu, lung tissue; Li, liver lymph node; Man, mandibularis lymph node; Par, parotid lymph node; A, pool A containing both medial retropharyngeal lymph nodes; B, pool B containing TraMedMes; ML, Matrixlysis;

Red deer animal 16 had a pathoscore of 4 in all investigated samples of this animal. It showed an open tuberculosis in the lung which was not examined in this study. Both sample pools of this animal were tested positive in all methods (Fig 2).

Red deer animal 17 only showed a pathoscore in pool C (sample 17 C) and a pathoscore of 0 in samples A and B. The observed pathoscore 1 corresponded to a single lesion of 2 mm in one of the two medial retropharyngeal lymph nodes. While samples A and B were negative in all tests, sample C was only positive by ML and the homogenate method, both resulting from the same starting material. This was the only tested animal which was culture negative in all samples and therefore would have been detected negative by routine diagnostic methods.

Red deer animal 18 showed similar results as red deer animal 17 with the difference that sample C was also positive by bacteriology and an occurrence of several milliar lesions in one medial retropharyngeal lymph node.

Red deer animal 19 presenting several "B" lesions of more than 2 cm in the tracheobronchial lymph node and in the lung, was only positive in sample pool C; containing both tissue samples of pathoscore 3 and 4, respectively. (In a very first test this animal was also positive in sample A, B and C by bacteriology.)

If comparing the ct-values of samples 17, 18 and 19 C, the detected ct-values of ML were always 4 to 5.9 ct-values lower than the ct-values of the homogenate method, which correspond a 16 to 60-fold higher sensitivity. Even the difference to the direct qPCR of sample 19C was remarkable. Nevertheless, pathoscore 0 samples of these three red deer animals were not detected by any other method than by ML.

Red deer animal 21 showed a single lesion of type “A” in the retropharyngeal lymph node. Bacteriology detected the lesions as well as NVL in sample B, while ML qPCR detected only the lesion containing sample material of the retropharyngeal lymph node. Direct qPCR was negative in both sample pools. The explanation for the negative ML qPCR might be an exhaust of sample material.

Vice versa red deer animal 22 showed a pathoscore of 3 and 0 in sample A and B, respectively (Fig 2). While sample A was detected by all methods, sample B with a pathoscore of 0 was only negative by bacteriology but positive by all other methods. The lowest ct-value of 25.4 of sample A was observed by direct qPCR due to its easy detection of a well-developed “B” lesion. As ML qPCR was performed from exhausting sample material, after material withdrawal for direct qPCR and bacteriology, the ct-value of ML qPCR, containing mainly lesions surrounding tissue, was higher than for direct qPCR. This was also observed for sample 1 and 9 of the pre-test. But in all these cases, the pathoscores were higher than 2. One or a few milliar lesions characterized as pathoscores 1 and 2 were always negative by direct qPCR (samples 17C, 18C, 21A and sample 4 of the pre-test). Only red deer sample 7 of the pre-test showed identical ct-values by both methods.

Some pathoscore 0 samples of red deer animals 20, 23, 24 and 26 were detected only by ML qPCR but no other methods indicating that ML qPCR is able to detect NVL samples. Only sample 25B was additionally positive by the homogenate method but with a much higher ct-value. NVL samples detected only by ML were samples 20A, 20B, 23A, 24B, 25B and 26B. As visible lesions were observed in other parts of the animals, these animals or samples cannot be classified as false positives.

As red deer animals 17 and 18, red deer animal 25 showed patho-morphologically visible lesions only in the one of the two medial retropharyngeal lymph nodes, but lesions of animals 23 and 25 were further developed and could be classified as "B" lesions. To avoid a dilution of the sample material A or B obligatory containing both medial retropharyngeal lymph nodes or the mediastinal- and tracheobronchial lymph nodes, respectively, material-containing lesions of these animals were cut out and treated separately as sample C. While red deer animals 17 and 18 were only positive by sample C (lesions containing material), ML qPCR additionally detected NVLs in sample A of animal 23 and sample B of animal 25.

The maximum amount of 10 g sample material was used in case of sample A of red deer animal 24. ML qPCR showed a ct-value of 35.2, direct qPCR a ct-value of 40 and the homogenate method no ct-value indicating that most of the lesion containing material of pathoscore 3 was exhausted and used for bacteriology which had to be repeated due to technical issues. To test, if more starting material always results in a lower ML qPCR ct-value, we performed ML of two different amounts of sample material in case of samples 20A, 20C and 25C. The hypothesis could be confirmed in case of sample 20C and 25C (Fig 2). The higher amounts of 6.3 g and 4.0 g compared to 2.2 g and 2.0 g, respectively, resulted in 3.3 and 4.5 lower ct-values. Our hypothesis could not be confirmed in the case for sample 20A where 7.2g sample material resulted in a higher ct-value than 2.6 g indicating an exhaust of sample material after many test repeats. In total, we were able to detect MTC in 35 out of 43 lymph nodes by ML qPCR which corresponds to 26 out of 26 positively "selected" animals. The detected lymph nodes also include six NVL lymph nodes. In comparison, bacteriology detected 28 out of 43 lymph nodes, which corresponds to 25 out of 26 positive animals. While bacteriology detected only one NVL sample (sample B of red deer animal 21), it did not detect sample C of red deer animal 17 and sample B of red deer animal 22, both classified as pathoscore 1 samples indicating that even the "gold standard" in TB diagnostic would have detected one red deer animal false negative. Direct qPCR detected 23 out of 43 lymph nodes corresponding to 21 out of 26 positive animals and the homogenate qPCR 25 out of 43 lymph nodes or 22 out of 26 positive animals. If these samples would have been tested in accordance to the national legislation RinderTbV 2008 in the current status, which says that only direct qPCR positive samples are further investigated by bacteriology in order to confirm the PCR positive result, five animals would have been diagnosed false negative by direct qPCR and four by the homogenate qPCR (Table 3A and 3B).

**Fig 3:**
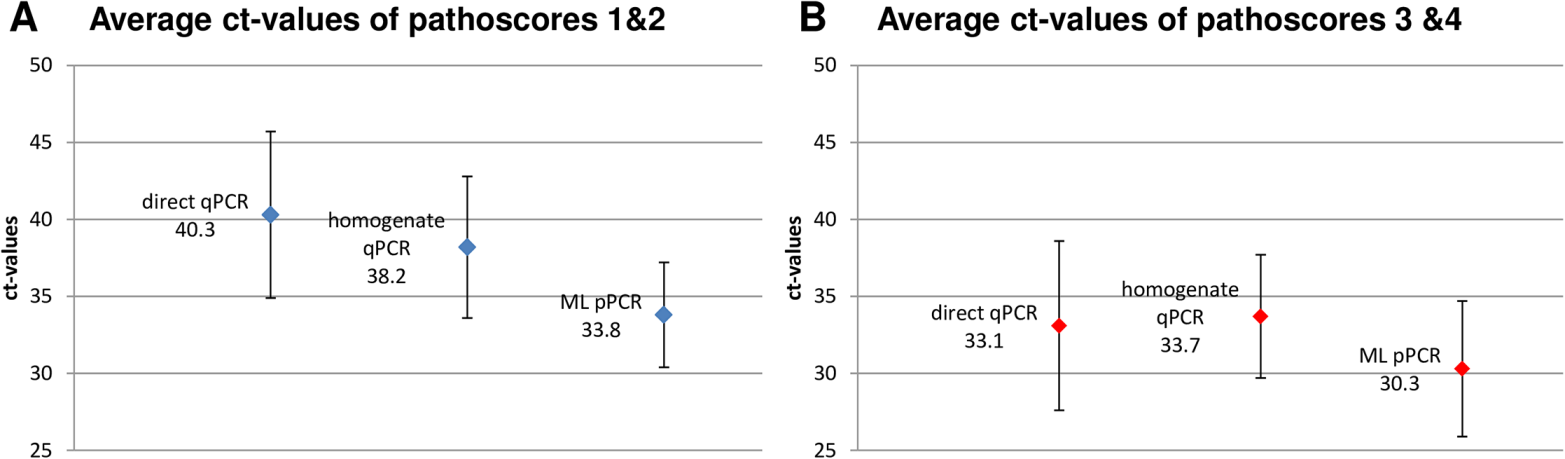
Average ct-values and deviations of pathoscore groups 1 & 2 (A) and 3 & 4 (B).

A correlation between ct-values and pathoscore group 1&2 (type “A” lesions”) and group 3&4 (type “B” lesions) is shown in Table 5 and Fig 3.

**Table 5:** Correlation between pathoscores and ct-values.

Method	pathoscore 1 & 2 average ct-value	Pathoscore 1 & 2 RSD	pathoscore 3 & 4 average ct-value	pathoscore 3 & 4 RSD
direct qPCR	40.3	5.4	33.1	5.5
homogenate qPCR	38.2	4.6	33.7	4.0
ML qPCR	33.8	3.4	30.3	4.4

RSD, relative standard deviation

For pathoscore group 1 & 2 the average ct-value of ML is 4.4 ct-values lower than the average ct-value of the homogenate qPCR and 6.5 ct- values lower than the average ct-value of the direct qPCR. For pathoscore group 3 & 4 the average ct-value of ML is 30.3 and therefore only about 3 ct-values lower than the remaining two qPCR methods, indicating an easier detection of well-developed lesions by direct and homogenate qPCR.

### Bacterial cultivation of ML derived bacteria (Viability test of ML)

To test, if the mycobacteria are still viable after ML, all samples of red deer animals and the cattle which derived from ML were grown on Loewenstein-Jensen and Stonebrink solid media; both containing PACT. After an incubation time of 12 weeks at 37°C, none of the 43 samples showed growth on either of the both media. On one hand this is a good result regarding the biosafety aspects, because due to very high bacterial loads there is no infection potential after the lysis step of ML. On the other hand without amplification of the pathogen, the lesion containing is limited and becomes more and more exhausted after every additional assay.

### Matrixlysis subtyping

**Fig 4:**
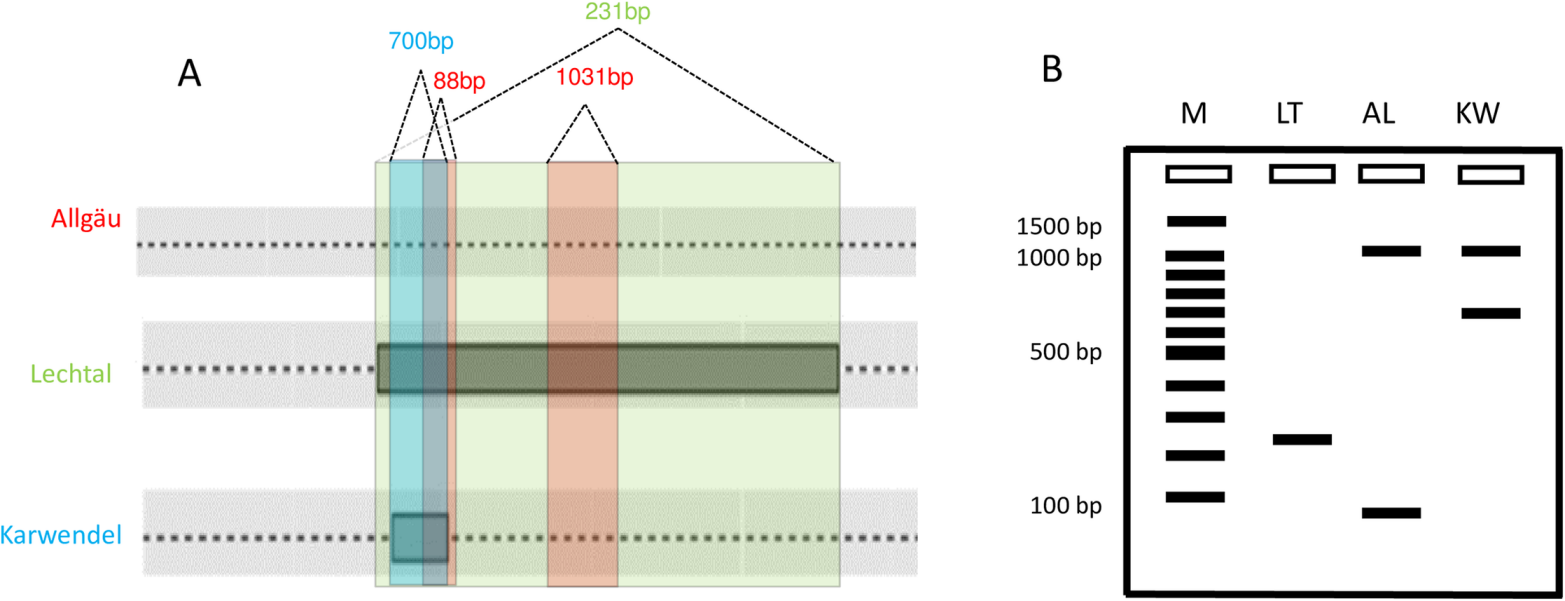
RD4 deletions (A) and classical PCR patterns (B) of alpine *M*. *caprae* subtypes Allgäu. Lechtal and Karwendel.

As qPCR can only give information if the pathogen is a member of the Mycobacterium tuberculosis complex (MTC), species identification and *M*. *caprae* subtyping or genotyping is used to be performed from bacterial culture isolates. Instead of *MIRU*-VNTR genotyping which are culture dependent, complex and extensive [25,26] we performed RD4 subtyping of our ML samples. In a previous study, whole genome data demonstrated that three *M*. *caprae* subtypes, ‘Allgäu’, ‘Lechtal’ and ‘Karwendel’ were characterized by genomic variations in the RD4. The three typical deletion patterns could be identified by PCR using bacterial culture (Fig 4) [15,16].

**Fig 5:**
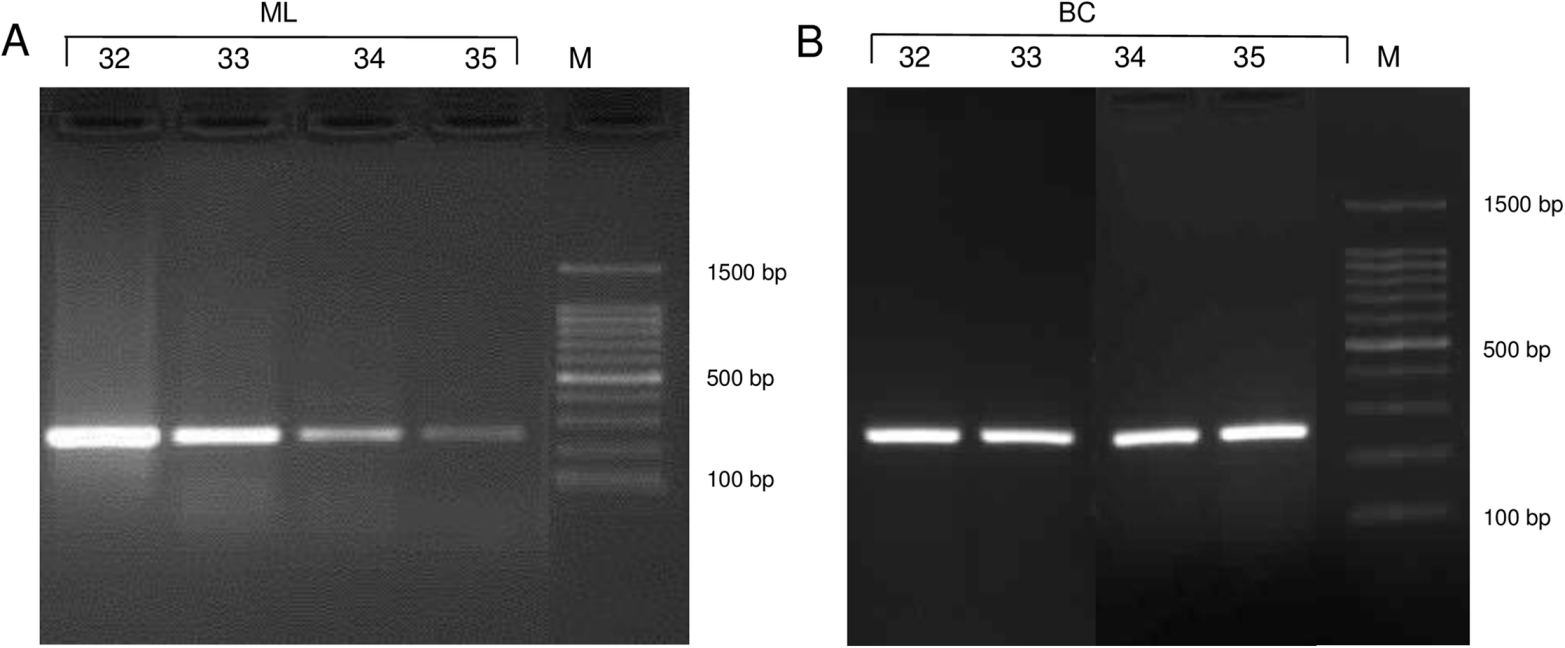
Identification of RD4 subtype Lechtal of red deer samples 32, 33, 34 and 35 derived from ML analysis (A) and bacterial isolates (B).

To test, if RD4 subtyping can be performed from ML resulting bacteria, we extracted the DNA of 12 red deer and six cattle ML derived bacteria pellets and performed RD4 subtyping. As 17 of 18 animals were confirmed by bacterial cultivation, RD4 subtyping was also performed from these 17 bacterial isolates and compared with results from ML subtyping. In order to test the DNA quality, all samples were tested by qPCR (Table 6). QPCR ct-values and the intensities of DNA bands on agarose gels of RD4 subtyping were well comparable. DNA concentration measurements performed with a Nanodrop did not accompany with the previous results. The reason for the different DNA concentration measurements could be the high amounts of host DNA in the samples. Apart from red deer samples 36, 38 and the cattle samples 41A, 43 and 44 all ML samples could be assigned to a subtype. ML samples 27 to 35, 37, 40, 41and 42 were identified as Lechtal subtypes (Fig 5 and S1 File).

**Table 6:** RD4 Subtyping of ML samples.

Sample No.	Host species	Lymph node/tissue	weight of lymph node for ML [g]	Pathoscore	RD4 Subtype (Result qPCR)	
					ML	Culture
27	Red Deer	Head	5.2	4	Lechtal (+)	Lechtal (+)
28	Red Deer	Head	6.5	3	Lechtal (+)	Lechtal (+)
29	Red Deer	Head	8.3	3	Lechtal (+)	Lechtal (+)
30	Red Deer	Head	6.7	3	Lechtal (+)	Lechtal (+)
31	Red Deer	Head	8.1	1	Lechtal (+)	no subtype (+)
32	Red Deer	Lung	3.7	5 (open Tb)	Lechtal (+)	Lechtal (+)
33	Red Deer	Lung	5.0	3	Lechtal (+)	Lechtal (+)
34	Red Deer	Lung	6.1	3	Lechtal (+)	Lechtal (+)
35	Red Deer	Lung	4.2	1	Lechtal (+)	Lechtal (+)
36	Red Deer	Lung	6.0	3	no subtype (+)	Lechtal (+)
37	Red Deer	Head	7.4	2 or 3	Lechtal (+)	culture neg
38	Red Deer	Re	3.9	3	no subtype (+)	Karwendel (+)
40	Cattle	Head	4.3	2 or 3	Lechtal (+)	Lechtal (+)
41	Cattle	Udder	4.8	2	no subtype (-)	Lechtal (+)
		Retro	4.2	2	Lechtal (+)	Lechtal (+)
42	Cattle	TrachMed	2.9	2	Lechtal (+)	Lechtal (+)
43	Cattle	Mediastinal	4.3	2	no subtype (+)	Lechtal (+)
44	Cattle	Lung	3.3	5 (open Tb)	no subtype (+)	Allgäu (+)

(+) qPCR positive; (-) qPCR negative; Re, one medial retropharyngeal lymph node; TrachMed, Tracheobronchial and Mediastinal lymph node; ML, Matrixlysis

Bacterial culture isolates from sample 31 did not show any subtype although qPCR showed a ct-value and the ML sample identified subtype Lechtal. ML samples 36 and 43 could not be subtypes although qPCR was positive and the bacterial culture isolates determined the Lechtal subtype. During the DNA extraction of these ML samples the protein precipitation did not work properly causing to leave some proteins in the mucous supernatant. Red deer sample 37 did not show any bacterial growth therefore no RD4 typing from the isolation could be performed. The corresponding ML sample detected the subtype Lechtal and was qPCR positive.

Although qPCR was positive and the bacterial culture could be classified as Karwendel subtype, it was not possible to subtype ML sample 38.

Sample material from the udder lymph node and the retropharyngeal lymph were used for subtyping cattle 41. RD4 subtyping revealed the Lechtal subtype from bacterial culture and ML, although subtyping from the ML udder lymph node was not possible. As qPCR of the same sample was negative too, we suggest that the sample material containing the gross lesions was exhausted already by methods from bacteriology.

Bacterial culture isolate of cattle sample 44 could be assigned to the Allgäu subtype. Although the ML qPCR was positive, it was not possible to identify its subtype.

Cattle sample 39 did not show any visible lesions after patho-morphological examination. This cattle was only positive by ante-mortem tests. In post-mortem routine diagnostic, all lymph nodes were negative by direct qPCR and bacterial culture. Bacterial growth was only observed when the three lymph nodes, the udder-, the intestinal- and the ileocecal lymph nodes, were pooled. ML RD4 subtyping was performed separately from all three lymph nodes. The intestinal lymph node could be subtyped as Lechtal by ML. ML samples from the udder and the ileocecal lymph nodes could not be assigned to a subtype and qPCR was negative suggesting that the intestinal lymph node was a NVL sample and the remaining two lymph nodes were negative (Table 7).

**Table 7:** RD4 Subtyping of sample pool.

Sample No.	Host species	Lymph node	weight of lymph node for ML [g]	Pathoscore	RD4 Subtype (Result qPCR)	
					ML	Culture
39	Cattle	Udder	1.5	0	no subtype (+)	Lechtal (+)
		Intestinal	5.5	0	Lechtal (+)	
		Ileocecal	4.7	0	no subtype (-)	

(+) qPCR positive; (-) qPCR negative

While Lechtal subtyping could be performed from almost all ML samples, subtyping of the Allgäu and the Karwendel subtypes was not possible in our study. Accompanying host DNA apparently is not a problem for ML qPCR detection but might be a problem for RD4 subtyping. A possibility to overcome this problem would be an additional purification step and the concentration of mycobacterial DNA from ML samples.

### ML next generation sequencing

Next Generation sequencing was used to study the detection sensitivity for *M*. *caprae* specific DNA in the presence of an excess of bovine DNA after enrichment of the mycobacteria by ML. NGS was performed for 3 samples using an Illumina Hiseq 1500 in a paired-end mode and dual indexing with a read length of 100 bp. Raw sequencing data were de-multiplexed using the Je's Illumina-illu [22] tool to reassign the reads to its respective samples. To ensure high quality data, no mismatch was demultiplexing allowed during and indices with quality scores below 30 were discarded. After demultiplexing an average number of 6.9 million reads per sample (Table 8) was obtained.

**Table 8:** Summary of the mapped reads of the 3 samples against the reference genome of *Mycobacterium caprae* and *Bos taurus* using BWA.

Sample No.	Total Reads produced by Illumina HiSeq	*B*. *taurus*	*M*. *caprae*
40	6567176	6418187	32
41	7039146	6891839	379
42	7269492	7110712	56

These reads were mapped against the reference genomes of *M*. *caprae* and *B*. *taurus* to determine the number of mapped reads. In overall, more than 99% of the reads were mapped to the *B*. *taurus* genome for all the three sequenced samples and only very few reads mapped to the *M*. *caprae* genome. The highest number of *M*. *caprae* specific reads (379) was found for sample 41 whereas a substantially lower number was found for samples 40 (32) and 42 (56). Percentage of duplicated reads in all the three ML samples is estimated as less than 1%.

## Discussion

To improve detection methods of Mycobacteria we have chosen ML as sample preparation method for enrichment of Mycobacteria in red deer and cattle tissue. The decision to use ML was made due to its ability to reduce tissue mass, concentrate the target and the ability to substitute commonly used detection methods like direct PCR and bacterial cultivation. A previously published modular study demonstrated solubilization of artificially spiked animal tissue [3] but so far ML was not performed using naturally tuberculosis infected samples. We could show, that ML preparation is suitable for qPCR detection and even more sensitive than bacterial cultivation additionally we received limitary results for RD4 subtyping and WGS.

Pathoscoring is a very helpful tool to classify lesions in the lung and in lymph nodes and additionally provides information about the infection stage of an animal. We used the pathoscore defined by Ballesteros *et al*. [19] which referred to post-mortem examinations of macroscopic lesions of European wild boars challenged with a *M*. *bovis* field strain. Even if lesions of different Tb host species are not completely identical, the described classification criteria reflect very well our pathomorphological observations in red deer and cattle.

Animals without visible lesions at all were mainly found positive in one of the two medial retropharyngeal lymph nodes by bacteriology suggesting a very early stage of infection [18]. By ML qPCR we even detected six NVL lymph node samples which were not detected by bacteriology indicating a much higher sensitivity than bacteriology. As all six red deer animals were detected positive by well-developed type “B” lesions in other tissues, a spread of bacteria might have always occurred to these lymph nodes without generating visible lesions there. So far, no PCR method performed directly from native sample material without additional processes was described to detect NVL samples. Although Courcoul *et al*. [27] reported a higher sensitivity for homogenate qPCR than for bacteriology, the relation of NVL samples to visible lesions was not specified. In our study, the sensitivity of bacteriology was higher than for direct qPCR or the homogenate qPCR. It must be emphasized, that bacteriology was performed with a different decontamination protocol as we used NALC instead of sulphuric acid. Depending on the decontamination procedure and the culture media, the percentage of detecting infectious material by bacteriology varied from 58% to 80% [28].

Cardoso *et al*. [29] estimated a similar sensitivity of bacteriology and PCR when investigating 35 lymph nodes of animals with macroscopic lesions only. These results are comparable with our direct qPCR which always detects visible lesions of a certain dimension (1 cm and more), but often missed milliar lesions of pathoscore 1 and 2 which were almost always detected by bacteriology and ML qPCR and in some cases, also by the homogenate qPCR. Contrary to clearly visible macroscopic lesions easily detected by direct qPCR, the homogenate qPCR shows a higher and therefore a more doubtful ct-value than direct qPCR due to the dilution factor of the starting material.

Due to the small amount of sample material used for PCR, it is very likely that PCR results in a false negative result mainly if detecting early stages of infection which do not present visible lesion or lesions of milliar sizes. The concentration of mycobacteria or mycobacterial DNA and subsequent PCR can solve this problem. Magnetic capture is an alternative method to ML. It allows enrichment and specific selection whether of the whole bacteria by anti-*M*. *bovis* antibodies [8] or by capturing MTC DNA using specific capture oligonucleotides for the PCR amplification of lysed sample material [7]. Stewart *et al*. [8] detected 2.7% of NVL lymph nodes by bacterial cultivation whereas 70.3% of the same samples tested *M*. *bovis* positive by the IMS-based tests, although the overall sensitivity of IMS-PCR (57,8%) performed directly from the native sample material was lower than for classical mycobacterial cultures (62.%) or IMS based MGIT (mycobacterial growth indicator tube) cultivation (68.2%). These results are comparable with ML results which detected seven out of 15 (46.7%) NVL lymph nodes while bacteriology detected only one out of 15 (6.7%). Magnetic capturing of MTC DNA detected 29 (29%) MTC positive cattle and red deer samples out of 100 while the homogenate qPCR detected only 23 (23%). Therefore, magnetic DNA capturing detected six cattle more which increased the number of positives from 10 to 16 out of 34 cattle. A discrimination between lesions containing and NVL samples was not performed and the results were only compared with homogenate qPCR but not with bacteriology or methods from pathomorphological inspections [7].

IMC but not ML or magnetic capture of MTC PCR facilitates cultivation of the selected mycobacteria on special growth media. Only 107 of 190 positive IMS -MGIT cultures could be confirmed by spoligotyping and the range of different *M*. *bovis* spoligotypes was less diverse than from classical cultivation, which was not surprising if considering that with the antibody used for magnetic beads selection of spoligotypes was performed. ML detected in almost all cases the *M*. *caprae* Lechtal subtype, but subtypes Allgäu and the Karwendel could only be determined by RD4 subtyping from bacterial cultures. Although it must be mentioned that due to its rare occurrence only one Allgäu subtype sample and one Karwendel subtype sample were available for our study.

ML enables the concentration but not the selection of specific MTC species or MTC DNA. IMC also detected NVL samples but spoligotyping provided limited results due to the selection of specific subtypes depending on the antibody used. Magnetic capturing of MTC DNA provides concentration and selection of MTC DNA but the percentage of NVL was not determined. Moreover, the sensitivity of this method was not compared to bacteriology, the gold standard. Furthermore, results were only evaluable if the sample material exceeded 3 grams. With ML one is not limited to a minimum or maximum amount of sample material and the method is also suitable for detecting NVL samples or samples with a few milliar lesions and therefore minimizes false negative results. Moreover, the method is cheap and uncomplicated but time consuming due to rather long incubation times. The main disadvantage compared to IM-PCR or magnetic capturing of MTC DNA is that samples which underwent ML contain also host species DNA. Further purification or selection procedures would provide a solution of this problem. Magnetic capturing of MTC DNA would be one possibility.

Next Generation sequencing of three ML samples has produced high throughput data with an average of 6.9 million reads per sample. Strict criteria for demultiplexing and quality scores of indexed reads ensured high quality of the sequence data and prevented errors due to the barcode misassignment. Mapping these high-quality reads against the reference genome of *M*. *caprae* and *B*. *taurus* revealed that more than 99% of the DNA content belongs to the host genome. *M*. *caprae* specific read count is found to be very low in all three ML samples with the maximum of 379 reads for the sample 41. In summary, the combination of Matrix Lysis and high throughput sequencing allowed the detection of mycobacterial DNA by moderate sequencing depth. Deeper sequencing or higher enrichment of mycobacterial DNA is required to characterize the pathogen.

## Supporting information

S1 File Supporting information regarding Fig 5. Unedited argarose gel photos shown in Fig 5.
(DOCX)
