# Supplementary material for: Matrixlysis, an improved sample preparation method for recovery of Mycobacteria from animal tissue material
Source: PLoS One. 2017 Jul 19;12(7):e0181157. doi: 10.1371/journal.pone.0181157 (PMC5517009; doi:10.1371/journal.pone.0181157)
Supplement: S1 File — Unedited argarose gel photos shown in Fig 5. (DOCX) [file pone.0181157.s001.docx]

DNA bands resulting from RD4 subtyping of samples 32 – 35 (from Bacterial culture and Matrixlysis) were clubbed into one agarose gel picture shown as Fig.5 in the revised manuscript.

**32 33 34 35** 36 37 M

1500 bp

500 bp

100 bp


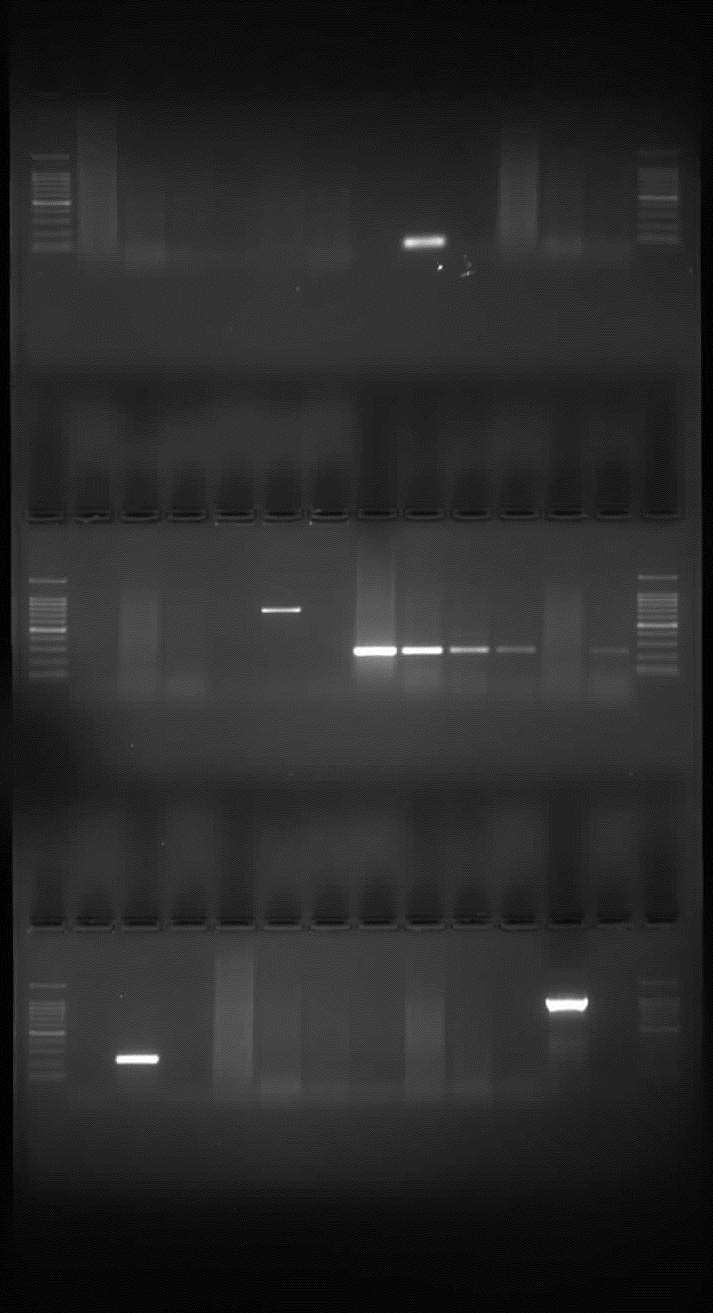


ML

M **34 35** 36 PC PC PC NC M

1500 bp

500 bp

100 bp


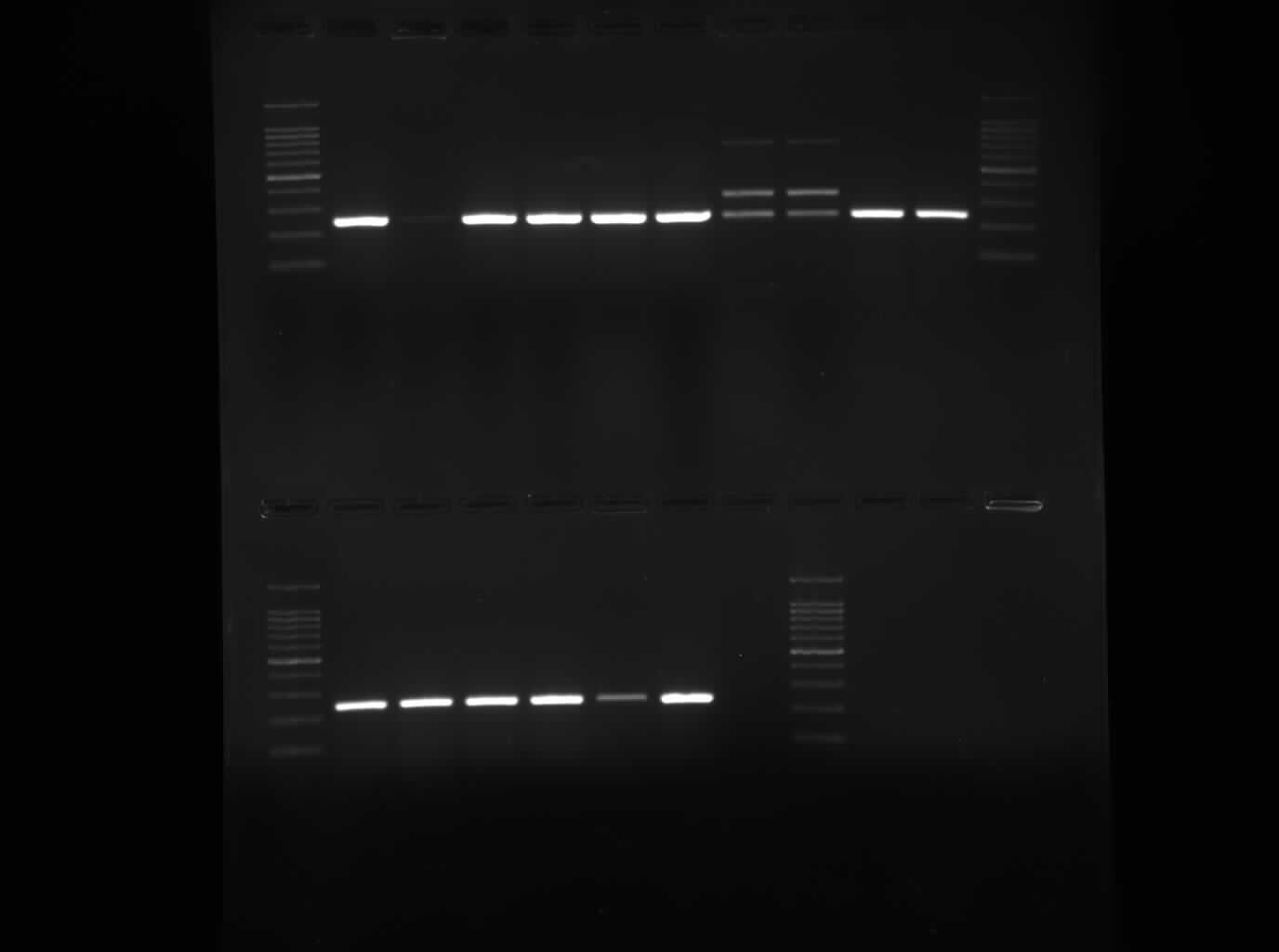


Bacterial Culture

M 42 73 72 71 75 76 78 78 **32 33** M

1500 bp

500 bp

100 bp


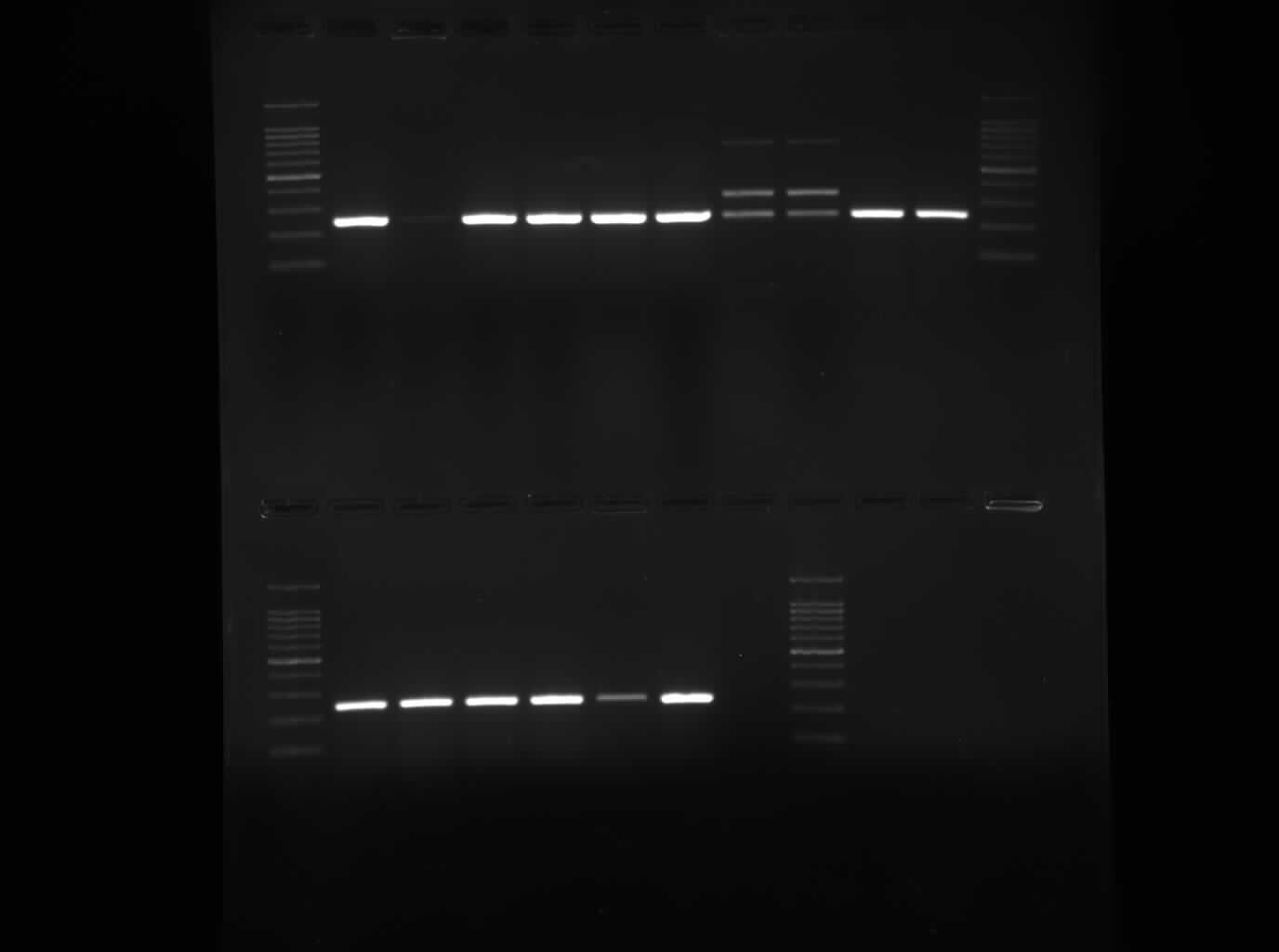


Bacterial Culture
